# Supplementary material for: Suppressive Effects of Lactobacillus on Depression through Regulating the Gut Microbiota and Metabolites in C57BL/6J Mice Induced by Ampicillin
Source: Biomedicines. 2023 Apr 1;11(4):1068. doi: 10.3390/biomedicines11041068 (PMC10135704; doi:10.3390/biomedicines11041068)
Supplement: Supplementary file 1 [file biomedicines-11-01068-s001.zip › 20220721 supplement Table S1.pdf]

Supplement Table S1 *L. plantarum* GMNL141 was determined by 16S rDNA sequencing and API150.

a

GMNL-141

GGCGTGCGGCGTGCTATACATGCAAGTCGAACGAACTCTGGT
ATTGATTGGTGCTTGCATCATGATTTACATTTGAGTGAGTGGC
GAACTGGTGAGTAACACGTGGGAAACCTGCCCAGAAGCGGG
GGATAACACCTGGAAACAGATGCTAATACCGCATAACAACTT
GGACCGCATGGTCCGAGTTTGAAAGATGGCTTCGGCTATCAC
TTTTGGATGGTCCCGCGGCGTATTAGCTAGATGGTGGGGTAA
CGGCTCACCATGGCAATGATACGTAGCCGACCTGAGAGGGTA
ATCGGCCACATTGGGACTGAGACACGGCCCAAACTCCTACGG
GAGGCAGCAGTAGGGAATCTTCCACAATGGACGAAAGTCTGA
TGGAGCAACGCCGCGTGAGTGAAGAAGGGTTTCGGCTCGTAA
AACTCTGTTGTTAAAGAAGAACATATCTGAGAGTAACTGTTCA
GGTATTGACGGTATTTAACCAGAAAGCCACGGCTAACTACGT
GCCAGCAGCCGGGGTAATACAA

REFERENCE: GMNL-141

DATE: 1/25/22

COMMENT:

VERY GOOD IDENTIFICATION	
Strip	API 50 CHL V5.2
Profile	- - - - + + - - - - - + + + + + - - - + + + - + + + + + + + [illegible] - -
Note	

Significant taxa	% ID	T	Tests against			
Lactobacillus plantarum 1	99.9	0.7	SBE 2%	TAG 7%		

Next taxon	% ID	T	Tests against			
Lactobacillus rhamnosus	0.1	0.23	LARA 8%	RHA100%	MDM 7%	MDG 85%
			MEL 9%	RAF 7%	GNT 85%	

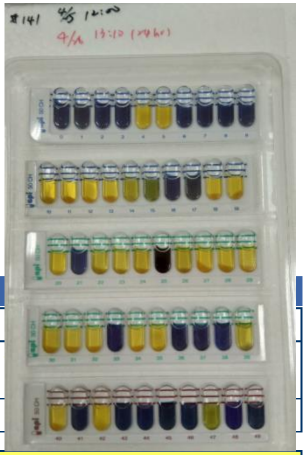

b

strips no.	carbohydrates substrate	GMNL-141	strips no.	carbohydrates substrate	GMNL-141	strips no.	carbohydrates substrate	GMNL-141
1	Glycerol	-	18	D-Mannitol	+	35	D-Raffinose	+
2	Erythritol	-	19	D-Sorbitol	+	36	Amidon (starch)	-
3	D-Arabinose	-	20	Methyl-α-D-mannopyranoside	+	37	Glycogen	-
4	L-Arabinose	-	21	Methyl-α-D-glucopyranoside	-	38	Xylitol	-
5	D-Ribose	+	22	N-Acetyl glucosamine	+	39	Gentiobiose	+
6	D-Xylose	+	23	Amygdalin	+	40	D-Turanose	+
7	L-Xylose	-	24	Arbutin	+	41	D-Lyxose	-
8	D-Adonitol	-	25	Esculin ferric citrate	+	42	D-Tagatose	+
9	Methyl-β-D-Xylopyranoside	-	26	Salicin	+	43	D-Fucose	-
10	D-Galactose	+	27	D-Cellobiose	+	44	L-Fucose	-
11	D-Glucose	+	28	D-Maltose	+	45	D-Arabitol	-
12	D-Fructose	+	29	D-Lactose (bovine origin)	+	46	L-Arabitol	-
13	D-Mannose	+	30	D-Melibiose	+	47	Potassium gluconate	-
14	L-Sorbose	+	31	D-Saccharose (sucrose)	+	48	Potassium 2-ketogluconate	-
15	L-Rhamnose	-	32	D-Trehalose	+	49	Potassium 5-ketogluconate	-
16	Dulcitol	-	33	Inulin	-			
17	Inositol	-	34	D-Melezitose	+			
