# Supplementary material for: Suppressive Effects of Lactobacillus on Depression through Regulating the Gut Microbiota and Metabolites in C57BL/6J Mice Induced by Ampicillin
Source: Biomedicines. 2023 Apr 1;11(4):1068. doi: 10.3390/biomedicines11041068 (PMC10135704; doi:10.3390/biomedicines11041068)
Supplement: Supplementary file 1 [file biomedicines-11-01068-s001.zip › 20221123 supplement Figure S1.pdf]

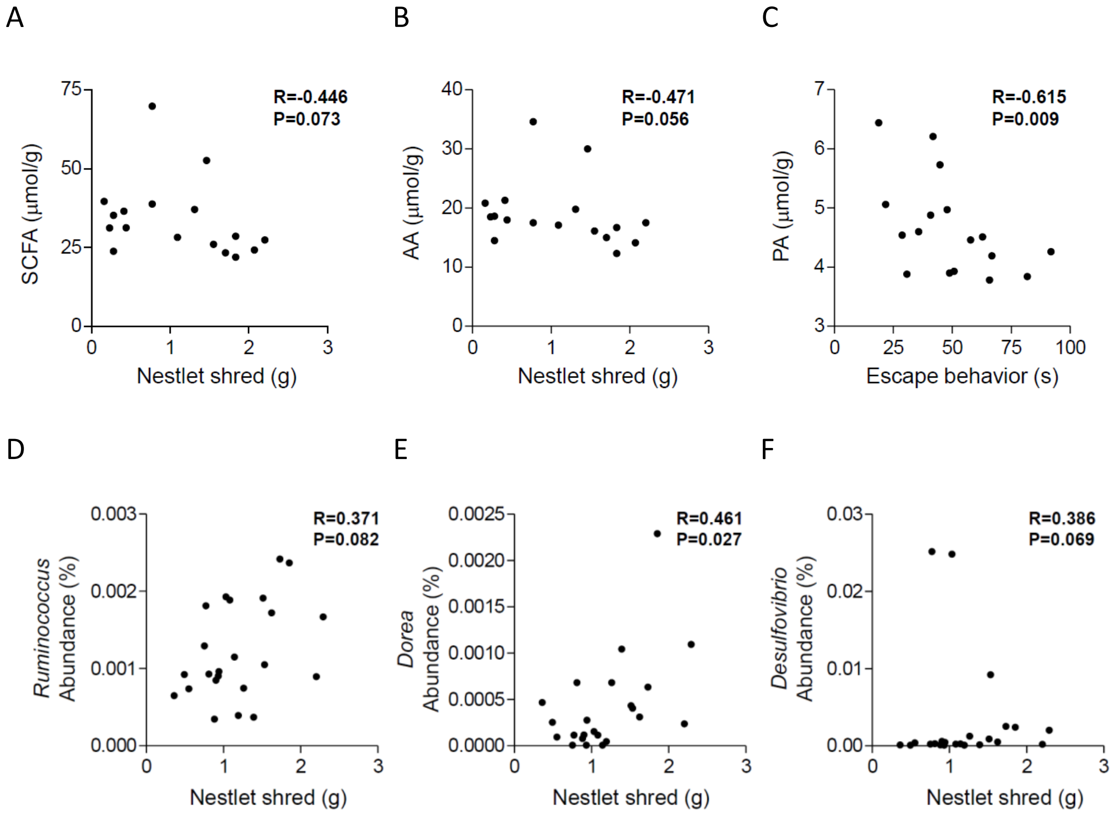

Supplement Figure S1

Correlations between the SCFA, gut microbiota and depression behaviors. Correlations between the variables were computed by Spearman's rho correlation provided by PASW Statistics 18 Software (SPSS Inc., Chicago, IL, USA). A p value less than 0.05 was considered statistically significant.
